# *In vitro* toxicity determination of antifungal constituents from *Combretum zeyheri*

**DOI:** 10.1186/s12906-016-1150-9

**Published:** 2016-06-02

**Authors:** Santana Mapfunde, Simbarashe Sithole, Stanley Mukanganyama

**Affiliations:** School of Pharmacy, College of Health Sciences, University of Zimbabwe, Mt. Pleasant, Harare Zimbabwe; Bio-molecular Interactions Analyses Group, Department of Biochemistry, University of Zimbabwe, P.O. Box MP 167, Mt. Pleasant, Harare Zimbabwe

**Keywords:** *Combretum zeyheri*, *Candida albicans*, *In vitro* toxicity, Phytoconstituents

## Abstract

**Background:**

*Candida albicans* is one of the organisms living on the human body symbiotically, but, in hosts with low immunity it becomes one of the most pathogenic fungal organisms. *Combretum zeyheri* has been reported to have antifungal, antibacterial and antioxidant activities. Medicinal plants are believed to be non-toxic by the general public. Toxicity studies, however, have indicated that they are capable of causing numerous side effects, therefore, evaluation of safety is required. The objective of this study was to determine the toxicity of the antifungal constituents of *Combretum zeyheri* on mammalian cells.

**Methods:**

Alkaloids, saponins, flavonoids-enriched extracts and crude ethanol extracts were prepared from the leaves of *Combretum zeyheri*. The broth microdilution method was used to investigate for antifungal activity, with miconazole used as the positive control. The MTT (3-(4,5-dimethylthiazol-2-yl)-2,5-diphenyltetrazolium bromide) assay was used to determine cell viability of the *Candida albicans* cells. The most potent extracts; the ethanol extract, alkaloids and saponins respectively, were further tested for their toxicity on sheep erythrocytes, mouse peritoneal macrophages and Jurkat T cells.

**Results:**

All *Combretum zeyheri* extracts displayed a dose-dependent antifungal activity and had IC_50_ values ranging from 16 μg/ml to 159 μg/ml for *Candida albicans*. The alkaloids, saponins and ethanol extracts were found to be non-toxic towards mouse peritoneal cells and Jurkat T cells. In the haemolysis assay, all extracts were haemolytic at varying degrees and showed their greatest haemolytic activity at the highest concentration of 5 mg/ml. The saponins were the least haemolytic, followed by the ethanol extracts and the alkaloids respectively. Although these extracts were haemolytic to some extent, they may considered safe at therapeutic concentrations since there was a large difference between the antifungal IC_50_ and haemolysis EC_50_ values_,_ hence a large therapeutic window.

**Conclusions:**

*Combretum zeyheri* antifungal constituents are, therefore, a potential source of lead compounds which can be developed into antifungal drugs of natural origin owing to *Combretum zeyheri*’s effective antifungal activity and low toxicity to mammalian cells.

## Background

Pathogenic fungi cause infections leading to critical threats on public health. The sudden uprising of fungal infections in the last few years is attributed to the population’s susceptibility to opportunistic infections precipitated by immune system suppression closely linked to malnutrition [1]. *Candida albicans* is one of the organisms living on the human body symbiotically, but, in hosts with low immunity it is one of the resilient fungal organisms [2]. *Candida albicans* causes a number of non-life threatening conditions including vaginitis and oral thrush [3]. The fungi can also pass through the blood stream and may affect systemic organs including heart valves and this may be fatal especially in immunocompromised individuals [4].

The emergence of mutant pathogenic strains that are resistant to current drugs, necessitates the need to find new, safe and effective drug compounds with minimum or acceptable side effects [4]. There are few effective antifungals available and most of them have unpleasant effects such as renal toxicity that occurs during the use of amphotericin B, and drug-drug interactions in the use of azoles [5]. The prevalence of fungal infections is increased by antibiotic resistance and antifungal drug toxicity especially after prolonged treatment, hence, there is need for new, safe and non-toxic drugs [1].

The World Health Organisation reports that greater than 80 % of the world’s population relies on traditional medicine to cater for primary health care [1]. Herbal medicines are believed to be free from side effects, less toxic and cheaper than conventional medicines. However, the cytotoxicity of plants-derived medicinal products has to be investigated [6]. A number of synthetic drugs were developed through mimicking the structures of compounds isolated from plants [7]. It is important to gain knowledge on the chemical constituents from plants to enable therapeutic agent discovery and unmasking other sources of such chemicals. Drugs from natural sources exhibit drug likeness and biological friendliness compared to synthetic products [8]. Plants synthesize bioactive compounds that are responsible for *in vitro* antimicrobial activity of plant extracts. These bioactive compounds include alkaloids, flavonoids, glycosides, saponins, terpenoids, tannins, carbohydrates and sterols [1].

The *Combretaceae* family has two genera and the largest and extensive genus, *Combretum,* consists of about 250 species distributed all through the tropics and subtropics of Africa, China and India [9]. The use of *Combretum* species in folk medicine include treatment of a large range of ailments including headaches, abdominal disorders, fever, gallstones, gastric ulcers, diarrhea, bilharzia, dysentery, hookworm, sore throat, pneumonia, urinary tract cleaning and conjunctivitis. Phytochemical studies on the *Combretum* genus have demonstrated the presence of various classes of phytoconstituents among them flavonoids, alkaloids, tannins and saponins [10]. *Combretum zeyheri* extracts have been shown inhibit ABC-type drug efflux pumps [4], and inhibit ergosterol synthesis in *C. albicans* [10].

Medicinal plants are believed to be non-toxic by the general public. However, toxicological studies indicate that they are also capable of causing numerous side effects, and therefore, the evaluation of their safety is warranted [11]. Cytotoxicity tests are crucial mainly to determine potential toxicity of compounds under study. Cytotoxicity may be determined using primary or transformed cells [12]. It is important to evaluate the efficacy and toxicity of plant constituents as chemical substances in plants may have haemolytic or anti-haemolytic effects on human red blood cells. The evaluation of cell membrane toxicity can be carried out on single cell models like erythrocytes and liposomes [13]. The objective of the study was to determine the toxicity of the antifungal constituents of *Combretum zeyheri* on mammalian cells.

## Methods

### Reagents, cell cultures and animals

All chemicals used in this study were from Sigma-Aldrich Co. (Steinheim, Germany). These included: diethyl ether, ethanol, n-butanol, sodium chloride, starch, Hank’s Balanced Salt Solution (HBSS), foetal bovine serum (FBS), penicillin, streptomycin, RPMI 1640 powder, sodium bicarbonate, trypan blue, acetic acid, ammonium hydroxide, methanol, 3-(4,5-dimethylthiazol-2-yl)-2,5-diphenyltetrazolium bromide (MTT), miconazole, ketoconazole, dimethyl sulfoxide, Sabouraud dextrose agar, Sabouraud dextrose broth, citric acid, sodium citrate dihydrate, D- glucose, peptone from vegetables, sulphuric acid, barium chloride, disodium hydrogen phosphate, potassium dihydrogen phosphate, potassium ferricyanide, potassium chloride, potassium cyanide, sodium bicarbonate and triple distilled grade water was used for all experiments. The fungus used, *Candida albicans* strain ATCC 10231 was a generous contribution from Prof K. Marobela (Department of Biological Sciences, University of Botswana). The use of animals and human cancer cell lines in this study was approved by the Joint Parirenyatwa Group of Hospitals and College of Health Sciences Research Ethics Committee (JREC/327/14, Harare, Zimbabwe). For the toxicity studies, Jurkat-T cells were and mouse peritoneal cells were used. Mouse peritoneal cells were extracted from 5 male Balb/c mice and sheep erythrocytes separated from sheep blood obtained from the Animal House, Department of Anatomy, (University of Zimbabwe Mt Pleasant, and Harare, Zimbabwe).

### Plant collection and preparation of extracts

*Combretum zeyheri* plant leaves were collected in Norton, Mashonaland West Province of Zimbabwe, geographical coordinates: 17.8833 ° S, 30.7000 ° E, and 1364 m above sea-level. The identities of the plants were authenticated by a botanist, Mr. Christopher Chapano, at the National Botanic and Herbarium Garden, Harare, Zimbabwe. Herbarium samples N6C7, were kept at the National Botanic and Herbarium Garden (Harare, Zimbabwe) and the Department of Biochemistry, University of Zimbabwe. The dried plant leaves were ground using a two speed blender (Cole Parmer instruments company, Vernon Hills, USA). All extractions were carried out according to Mangoyi et al. [7], with a few modifications. The dried leaves of *C. zeyheri* were ground to a fine powder in a blender (Philips co., Shanghai, China). To fifty grams of powder, 500 ml of ethanol was added. Extractions were allowed to proceed overnight. The extract were filtered using Whatman No.1 filter paper. The ethanol extract was dried using a rotovaporator (Rotovapour RII, BUCHI, Flawil, Switzerland). The extract were collected, weighed and stored in the dark at room temperature.

### Preparation of the alkaloid-enriched fraction

Acetic acid (20 ml) in 180 ml of ethanol was added to 50 g of the powdered leaf sample, covered and left to stand for 4 h. The mixture was filtered and the filtrate concentrated on a water bath (Rotovapour RII, BUCHI, Flawil, Switzerland) to 25 % its original volume. Concentrated ammonium hydroxide was added drop by drop to the concentrated extract until precipitation was complete. The solution was left to settle then 9 % ammonium hydroxide was used to wash the precipitate. The residue was dried under a stream of air from a fan and weighed.

### Preparation of the flavonoid- enriched fraction

Fifty grams of powdered leaf sample was extracted twice with 80 % methanol (40 ml distilled water + 160 ml methanol) [9]. Whatman filter paper No. 1 was used for filtration and the filtrate was evaporated on a water bath until dry and at constant weight. Twenty grams of dried sample was mixed with 20 % ethanol and put in an orbital shaker for 30 min. The mixture was heated at 55 °C for 4 h over a water bath. The residue was re-extracted with 20 % ethanol and filtered. The filtrate was then extracted twice with diethyl ether, the ether layer was discarded and the aqueous layer retained. N-butanol was added to the aqueous layer and a pinch of sodium chloride was added to withdraw any excess water. The resulting solution was filtered and the filtrate oven dried at 40 °C until a constant weight was obtained.

### Preparation of a saponin-enriched fraction

The method used for the extraction was adapted from Hostettmann et al. [14]. A mass of 50 g was weighed and extracted in 200 mls of 70 % ethanol for 24 h and this was done for 3 times at room temperature. The mixture was then filtrated using Whatmann filter paper number 1. The combined ethanol solutions were concentrated to a small volume using a Buchii Rotavapour 11 and extracted 3 times in succession with 200 mls chloroform for 24 h. The resultant solution was mixed with an equal volume of n-butanol and allowed for solvent-solvent extraction for overnight. The n-butanol layer was then concentrated to dryness to give the purified saponin fraction.

### Preparation of *C. albicans* cell cultures

Sabouraud dextrose broth (SDB), was prepared and sterilized by autoclaving. Five hundred microliters of *Candida albicans* cells from glycerol stocks were incubated in 20 ml of the media. The negative control contained 20 ml of media. The cells were incubated at 37 °C for 24 h, in an orbital shaking incubator at 120 rpm. The cells were then cultured on Sabouraud dextrose agar (SDA) plates. A single colony from the agar plates was incubated in broth at 37 °C, for 24 h, in a shaking incubator at 120 rpm (Labcon, Jeicho, Korea). The concentration of the *C. albicans* cells was calculated in relation to the 0.5 McFarland standard. The cells were diluted to 2 × 10^6^ cfu/ml for use in the antimicrobial susceptibility assays.

### Determination of growth inhibition parameters

Determination of growth inhibition parameters was done using MTT (3-(4,5-dimethylthiazol-2-yl)-2,5-diphenyltetrazolium bromide) as described by Eloff and McGaw, [15]. Samples of the extracted phytoconstituents were dissolved in dimethyl sulphoxide (DMSO) to make 10 mg/ml solutions which were serially diluted with 10 % DMSO then further diluted with media to give solutions with a 2 % DMSO final concentration. Test samples (100 μl per well) were pipetted onto round bottomed 96-well plates. Cultures of the *C. albicans* were transferred into fresh nutrient broth to make a 2 × 10^6^ cfu/ml cell suspension, 100 μl aliquots of the fresh culture were added to the wells. The final DMSO concentration used in the study was 1 %. The positive control used was miconazole and an appropriate solvent blank served as the negative control. The microplates were incubated at 37 °C at 100 % relative humidity for 24 h. To determine cell viability, 25 μl of 2 mg/ml MTT were added to all wells on the 96-well microplates and incubated for three hours. MTT is converted to purple, insoluble formazan crystals in the presence of metabolically viable cells. DMSO was used to dissolve the crystals and the intensity of the purple colour was spectrophotometrically measured at 590 nm using a microplate reader (Tecan Genios-Pro microplate reader, Grödig, Austria)*. C. albicans* from the wells were sub-cultured on agar and incubated at 37 °C for 24 h, growth observed.

### Toxicity determination using the haemolysis assay

The haemolysis assay was determined as described before [16]. A mass of 0.162 g sodium citrate was weighed and placed in a conical flask. Sheep blood (50 ml) was aseptically collected and placed in the flask containing the sodium citrate. An equal volume of Alsever solution was immediately added. The blood was centrifuged at 3000 rpm for 10 min. The supernatant was discarded then the residue was washed three times with a 1:5 volume of phosphate buffer saline (PBS), by centrifuging at 4000 rpm for 5 min in a Hettich Rotofix 32 centrifuge (Tuttlingen, Germany). The supernatant was discarded. The cells were diluted four fold with PBS and the resulting suspension was used for determination of haemolysis. The erythrocyte suspension (500 μl) was incubated with 500 μl of the test sample extract in phosphate buffer saline (PBS) pH 7.2 for 90 min at 37 °C. After incubation the tubes were spun in a microcentrifuge (Hermle Z 232 M-2, Wehingen, Germany) at 3000 rpm for 1 min. The resulting supernatant (200 μl) was added to 3 ml of Drabkin’s reagent (0.2 mg/ml pottasium ferricyanide, 0.1 mg/ml potassium cyanide and 1 mg/ml sodium bicarbonate). The positive control consisted of 500 μl un-centrifuged mixture of erythrocyte suspension and 500 μl of buffer from which 400 μl was added to 3 ml Drabkin’s reagent (to obtain 100 % hemolysis). The negative control was used to measure the level of spontaneous hemolysis and this was made by mixing 500 μl of erythrocyte suspension and 500 μl of buffer, centrifuging at 3000 rpm for 60s then adding 200 μl of the supernatant to 3 ml of Drabkin’s reagent. Aliquots (200 μl) of the supernatants in Drabkin’s reagent were placed on round bottomed 96-well plates. To determine the amount of haemoglobin released, the absorbance of samples at 590 nm were read using a Tecan Genios Pro microplate reader (Grödig, Austria). The percentage hemolysis for each sample was calculated according to the equation:

$$ \mathrm{Percentage}\ \mathrm{hemolysis} = \left(\mathrm{sample}\ \mathrm{absorbance}/\mathrm{positive}\ \mathrm{control}\ \mathrm{absorbance}\right) \times 100. $$

### Toxicity tests on mouse peritoneal cells

To increase peritoneal cells yield, 1 ml of starch solution (20 %) was injected intraperitoneally into 5 male Balb/c mice (31 ± 3 g) and left for 24 h [16]. Each mouse was euthanized by cervical dislocation. The mouse was then sprayed with 70 % ethanol and mounted on a styrofoam board on its back. The outer skin of the peritoneum was cut using scissors and forceps and gently pulled, exposing the inner peritoneal skin. Cold PBS with 3 % FBS (5 ml) was injected into the peritoneal cavity taking care not to puncture any organs. The peritoneum was then massaged to remove any attached cells into the PBS. A 25 g needle was inserted, bevel up and attached to a 10 ml syringe in the peritoneum to collect as much fluid as possible. The collected fluid was put into tubes kept on ice. An incision in the inner skin was made to collect the remaining peritoneal fluid from the cavity. Samples with visible blood contamination were discarded. The collected suspension was centrifuged for 10 min at 1500 rpm in a Hettich Rotofix 32 centrifuge. The supernatant was discarded and the cells were resuspended in RPMI and incubated overnight at 37 °C in a 5 % CO_2_ Shel lab incubator (CO_2_ series Sheldon Mfg. Inc, Cornelius, USA). Cells were exposed to 0.4 % trypan blue and were manually counted using a haemocytometer counting chamber under a Celestron digital light microscope (Celestron, Los-Angeles, USA) using a × 10 objective lens. The *C. zeyheri* ethanol extract, saponins and alkaloids-enriched fractions were diluted such that the lowest concentration was the IC_50_ for antifungal activity and the final concentration of DMSO was 1 %. The cells were incubated in 96-well plates in the presence of extracts for 24 h while another set of plates was incubated for 72 h at 37 °C in a 5 % CO_2_ Shel lab incubator (Sheldon Mfg. Inc., Cornelius, USA). Each well contained 30 μl of the test substance, 154 μl of RPMI and 46 μl of 0.5 × 10^5^ cells/ml. After the 24 h the optical density was measured on a Tecan Genios-Pro microplate reader at 590 nm. After the 72 h incubation the cells were stained with 0.4 % trypan blue dye and counted using a haemocytometer counting chamber under a Celestron digital light microscope.

### Toxicity tests on Jurkat T cells

Cells stained with 0.4 % trypan blue were manually counted using a haemocytometer counting chamber under a Celestron digital light microscope (Celestron, Los-Angeles, USA) to determine cell density using an objective lens at × 10 magnification. The *C. zeyheri* ethanol extract, saponins and alkaloids-enriched fraction were diluted such that the lowest concentration was the IC_50_ for antifungal activity and the final concentration of DMSO was 1 %. The cells plated in 96-well plates in the presence of extracts were incubated for 48 h at 37 °C in a 5 % CO_2_ Shel lab incubator. Each well contained 30 μl of the test substance, 181 μl of RPMI and 19 μl of 1 × 10^5^cells/ml. After the 48 h incubation 25 μl of MTT was added and incubated for 4 h. A volume of 50 μl DMSO was added and optical density was measured on a Tecan Genios-Pro microplate reader at 590 nm.

### Statistical analysis

One-way analysis of variance test (ANOVA) with Dunnett’s Multiple Comparison Post Test was used to analyse the results. All columns of treatments were compared to the control. The values with a p-value < 0.05 or less were considered statistically significant. Graphical and Statistical analyses were carried out using Graphpad Prism 5® Software (Version 5.0, Graph pad Software Inc, San Diego, USA).

## Results

### Extraction of plant phytoconstituents

Leaves are the phytoconstituents manufacturing site in a plant, therefore, the phytoconstituents are found in varying amounts. For this plant leaf components, flavonoids appear to be the most abundant phytoconstituents in the *C. zeyheri* leaves followed by alkaloids and saponins respectively as shown in Table 1.

**Table 1:** Mass and percentage yield of the extracted samples from *Combretum zeyheri* leaves

Sample	Mass	Percentage yield
Flavonoids	5.341 g	10.68 %
Ethanol extract	3.106 g	6.212 %
Alkaloids	1.562 g	3.124 %
Saponins	0.082 g	0.410 %

The masses are from a starting sample of 50 g of the leaf extracts. The percentage yield was calculated as a percentage of the starting mass

### Antifungal activity

**Fig. 1:**
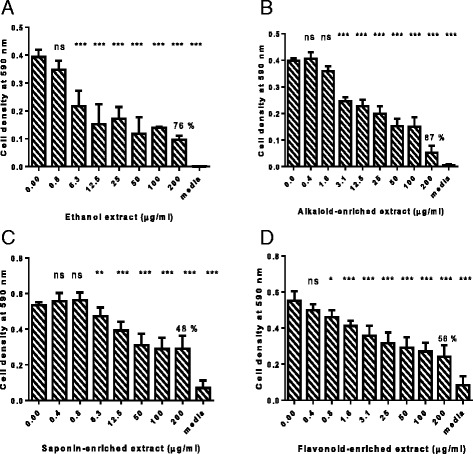
Antifungal effects of *C. zeyheri* extracts on *C. albicans* growth, where (**a**) is the ethanol extract, (**b**) the alkaloid-enriched fraction, (**c**) the saponin-enriched fraction and (**d**) the flavonoid-enriched fraction. Concentrations are in μg/ml and asterisks represent growth that is significantly different from the control (* = *p* < 0.5, ** = *p* < 0.001 and *** = *p* < 0.0001) ANOVA with Dunnet’s multiple comparison test. Values are for mean ± SD for *n* = 4. Percentage values on highest concentration indicate percentage decrease in *C. albicans* growth

**Fig. 2:**
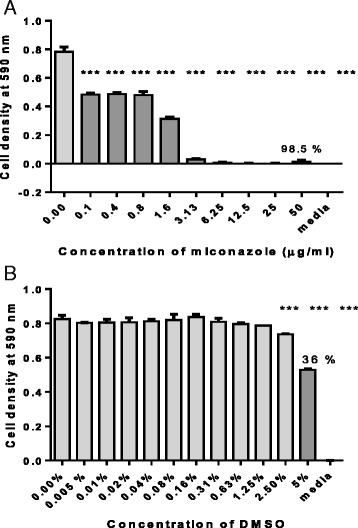
The effects of (**a**) miconazole and (**b**) solvent DMSO on *C. albicans* growth. Asterisks represent growth that is significantly different from the control (* = *p* < 0.5, ** = *p* < 0.001 and *** = *p* < 0.0001) ANOVA with Dunnet’s multiple comparison test. Values are for mean ± SD for *n* = 4. Percentage values on highest concentrations indicate the percentage decrease in *C. albicans* growth

The antifungal activity of *C. zeyheri* ethanol extract, alkaloids, flavonoids and saponins was determined by the broth microdilution method. All extracts were shown to possess a dose-dependent antifungal activity (Fig. 1). However, the highest concentration of 200 μg/ml, used in the experiments was too low to obtain the MIC and MFC values, hence, the IC_50_ was determined instead. The phytoconstituents with the lowest IC_50_ value and consequently the greatest antifungal activity were the alkaloids, although the crude ethanol extract had greater antifungal activity. IC_50_ values are shown in Table 2. Miconazole was used as the control drug, with an MIC of 6.25 μg/ml and the final DMSO concentration of 1 % had no effect on *C. albicans* growth as illustrated in Fig. 2.

**Table 2:** MIC and IC_50_ values determined for the phytochemical constituents

Sample	MIC	IC_50_
Ethanol extract	-	16 μg/ml
Alkaloid-enriched fraction	-	20 μg/ml
Saponin-enriched fraction	-	100 μg/ml
Flavonoid-enriched fraction	-	159 μg/ml
Miconazole	6.25 μg/ml	1.1 μg/ml

The IC_50_ was determined as the concentration that caused a reduction in 50 % of the growth based on the absorbance value at 590 nm of the positive growth control sample. The minimum inhibitory concentration (MIC) is the lowest concentration of an antimicrobial that will inhibit the visible growth of a microorganism. (-) indicates that no MIC was obtained

### Heamolysis assay

**Fig. 3:**
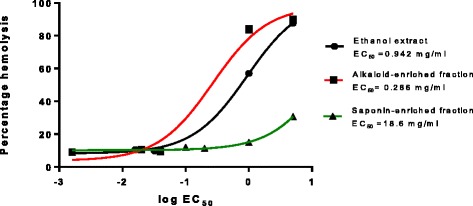
The hemolysis of sheep erythrocytes induced by exposure to the ethanol extract, alkaloid and saponin –enriched fraction. A plot of log EC_50_ versus percentage hemolysis were used to determine the concentration of the extract that caused a 50 % reduction in hemolysis. The percentage hemolysis for each sample was calculated according to the equation: Percentage hemolysis = (sample absorbance/positive control absorbance) × 100. Values are for mean ± SD for n = 6

**Fig. 4:**
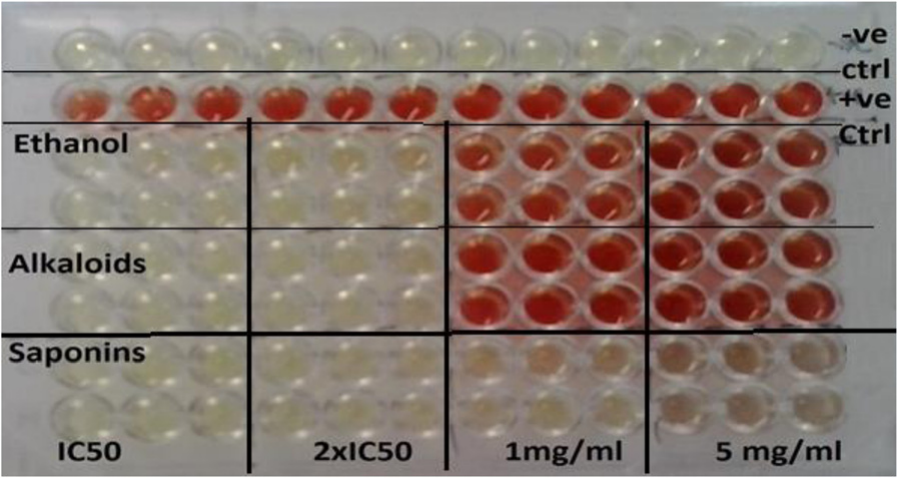
Image showing a 96-well plate for hemolysis of sheep erythrocytes exposed to *C. zeyheri* ethanol extract, alkaloid-enriched fraction and saponin-enriched fraction. The negative control (−ve ctrl), was a measure of spontaneous hemolysis and contained PBS and erythrocytes only, in Drabkin’s reagent and the positive control (+ve ctrl) which shows 100 % hemolysis was obtained by mixing un-centrifuged erythrocytes in PBS with Drabkin’s reagent

There was increased heamolysis as the sample concentration increased. The alkaloids exhibited the greatest hemolytic activity while, saponins were the least haemolytic (Fig. 3). The EC_50_ is the concentrations that caused 50 % hemolysis of the sheep erythrocytes (EC_50_). The ethanol extract showed an EC_50_ of 0.942 mg/ml, the alkaloid-enriched fraction a value of 0.286 mg/ml, and the saponin-enriched fraction a value 18.6 mg/ml. Thus, the saponin –enriched fraction was the least toxic to the erythrocytes. The varying color intensity of the lysed erythrocytes in Fig. 4 show that hemolysis was dose-dependent.

### Effects of phytoconstituents on mouse peritoneal cells and Jurkat T cells

**Fig. 5:**
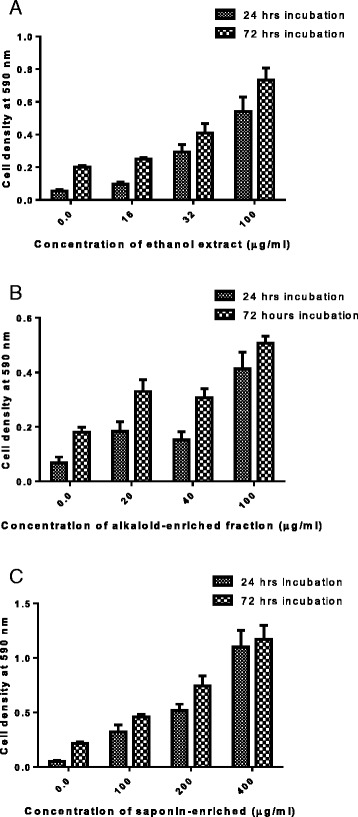
The effects of *C. zeyheri* (**a**) ethanol extract, (**b**) alkaloid-enriched fraction and (**c**) saponin-enriched fraction on mouse peritoneal cells. The graphs are a comparison of the cell density measured at 590 nm after 24 and 72 h of incubation Concentrations are in μg/ml. Values are for mean ± SD for *n* = 24

**Fig. 6:**
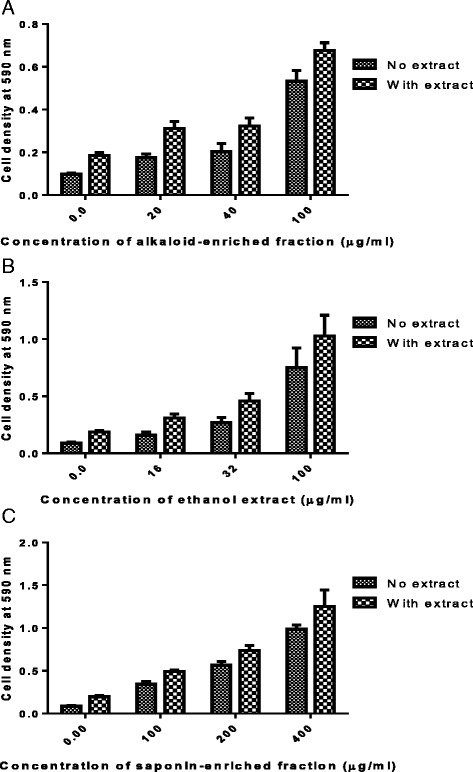
The effects of *C. zeyheri*, (**a**) alkaloid-enriched fraction, (**b**) ethanol extract and (**c**) saponin-enriched fraction on Jurkat T cells. A comparison of the cell density at 590 nm in the absence and presence of an extract. Concentrations are in μg/ml. Values are for mean ± SD for *n* = 24

Toxicity of *C. zeyheri* phytoconstituents was tested on mouse peritoneal cells. All test samples were non-toxic to the peritoneal cells. Moreover, they showed a dose-dependent increase in mouse peritoneal cells proliferation. Additionally, the proliferation of the peritoneal cells also increased with increasing time (Fig. 5). Similar to the mouse peritoneal cells, the *C. zeyheri* constituents promoted the growth of Jurkat T cells. The extracts were all shown to be non-toxic to the Jurkat T cells (Fig. 6).

## Discussion

Of late there was a reluctance in pursuing plants as medicine sources due to the evolution of synthetic antimicrobials. These synthetic drugs were effective for some time until the sudden uprising of a number of limitations to their use. Research has, thus, focused on sources of antimicrobials from ethnobotany and ethnopharmacology [17]. The number of people with suppressed immune systems is escalating and this has been associated with the development of resistant fungal species, which is the leading reason why there is need to develop new effective and safe antifungals [18].

This study showed that *C. zeyheri* possesses antifungal activity against *Candida albicans* and this is in agreement with other previous studies [18–20]. The ethanol extract, alkaloid, saponin and flavonoid-enriched fractions exhibited antifungal activity. This is in agreement with the work done by Mangoyi et al. [7], who reported that alkaloids and saponins had anticandidal effects, while flavonoids were not active despite them being the most abundant phytoconstituents among those extracted from *C. zeyheri* leaves. The ethanol extract was the most active and this could be because it contains a combination of many phytoconstituents that augment each other’s effects. Consequently, it is important to separate, purify, identify and determine the characteristics of the biomolecules in order to identify the compounds responsible for the antifungal activity of the plant extracts [21]. In this study the alkaloids, flavonoids and saponins were extracted as a preliminary step towards identifying the specific active constituents. Indeed it has been shown that the flavonoid 5-hydroxy-7,4’-methoxyflavone isolated from *C. zeyheri* had antifungal effects [20]. The MIC of the positive control, miconazole was as expected and was found to be at 6.25 μg/ml. Some authors have suggested that the sensitive concentration to miconazole for *C. albicans* should be ≤ 8.0 μg/ml [22]. The solvent used, DMSO increases membrane permeability within the plasma membranes of cells. Other investigations have shown that DMSO may affect cell growth, suggesting that it has the ability to lessen or potentiate inhibitory activities of water soluble drugs using the standard susceptibility assays [23]. These reports necessitated the need to investigate the effects of DMSO on *C. albicans.* At a concentration of 1 %, DMSO had no growth inhibitory effects on the growth of *C. albicans*.

The investigation of haemolytic activity is used as a cytotoxicity measure and may be used to roughly calculate therapeutic index for antimicrobials [24]. Determination of hemolysis activity is an alternative screening process for evaluating simple toxicity. It is quick, reproducible and cheap to carry out, enabling the reduction of laboratory animals for *in vivo* studies, therefore, reducing, refining and replacing studies carried out on animals [25]. Hemolysis is a result of membrane lipid bilayer lysis causing red blood cell destruction [26]. Haemolytic activity of the *C. zeyheri* constituents was shown to be concentration-dependent. This is in agreement with the work produced by Noudeh et al. [13] who showed that hemolytic activity increases with increasing extract concentration based on Fick’s law. Diffusion flux from a membrane is proportional to differences in concentration on both sides, therefore, increasing extract concentration in the extra membrane causes the extract to diffuse to the intra membrane until it gets to a certain concentration, leading to membrane damage and hemolysis. It should be noted that the haemolytic activity of an extract depends on chemical composition of the extract as illustrated by the different haemolytic activities of the *C. zeyheri* ethanol extract, alkaloid and saponin-enriched fractions. The alkaloid-enriched fraction showed the most haemolytic effects followed by the crude ethanol extract and the saponin-enriched fraction showed the least haemolytic activity. Although the ethanol extract possibly contains various phytoconstituents, it had a lesser haemolytic effect than the alkaloid-enriched fraction. We suggest that the crude ethanol extract may contain other phytoconstituents that are anti-haemolytic. Inalegwu and Sodipo, [27] indicated that the haemolytic activity of crude extracts can be attributed to saponin content. In contract in this study, saponins had the least haemolytic effect. Noudeh et al. [13], however, pointed out that although saponins naturally possess cell membrane permeabilising activity, their haemolytic activity depends on the chemical composition. Triterpenoid aglycon saponins are less haemolytic compared to saponins with steroidal aglycons. It is, therefore, plausible to suggest that the *C. zeyheri* saponins might possess the triterpenoid aglycons. Compared to the IC_50_ values, the haemolysis effective concentration (EC_50_) values of the potent phytoconstituents were much higher, thus, giving a large therapeutic window for the constituents as reported by Cherion et al. [28]. In this study, alkaloids were shown to have the greatest hemolytic activity but with a lower antifungal activity against *C. albicans*, compared to the ethanol extract. It may be suggested that use of an ethanol extract in antifungal treatment is better because once there is separation of the whole extract to phytoconstituents, there would be decreased antifungal activity, while simultaneously increasing haemolysis for some of the phytoconstituents, notably the alkaloids.

First line immunological defense versus bacterial, fungal and viral infections and tumour cells involves macrophages and natural killer (NK) cells. Macrophages also present antigens to lymphocytes during specific immunity development and in addition release factors that aid lymphocyte activity [29]. The peritoneal cavity in the abdomen of mammals is membrane bound and filled with fluid, in which the gastrointestinal tract, spleen and liver are housed. It is also a favourable site for the collection of naïve macrophages because they are found in large numbers. Peritoneal cavity cells are ideal for use in studies pertaining to different immune cells particularly macrophages [30]. The *C. zeyheri* ethanol extract, alkaloid and saponin-enriched fractions were not toxic to the mouse peritoneal cells but instead promoted the growth and survival of these cells. The extracts could be acting as antigens which may be triggering an immune response, hence, the mouse peritoneal cells’ proliferation. This is in agreement with the work of Bukowski et al. [31], who showed that immune system cells, specifically T lymphocytes can proliferate when exposed to some antigenic alkylamines. These alkylamines are mostly found in bacterial supernatants but are also present in some plants for example as tea and apple constituents. There is a possibility that *C. zeyheri* leaf extracts contain these substances. Other plant extracts, for example Korean mistletoe lectin, were also shown to increase peritoneal macrophages and splenic natural killer cells’ activity [29]. This activity was shown to be related to their *in vivo* action of the immune system, where lectin treatment increased the number of macrophages and lymphocytes in the peritoneal cavity of mice [32]. *Allium sativum* extracts enhance immune function by increasing the number of macrophages and T lymphocytes [33, 34]. Extracts from *A. precatorius* were also found to be non-toxic to mouse peritoneal macrophages [35]. Leaf extracts from *C. zeyheri* can, thus, are, thus, non-toxic to mouse peritoneal cells. Their growth-enhancing effect would be beneficial to immunocompromised patients if taken as herbals from *C. zeyheri* since the extract would act as an antifungal and immunity booster at the same time.

Jurkat cells are a cancer cell line which was established from the peripheral blood of a 14 year old boy who had T cell leukaemia [36]. These cells allow for *in vitro* testing in studies as they are capable of being cultured and proliferate under appropriate conditions [37]. There was no toxicity reported when Jurkat T cells were exposed to *C. zeyheri* ethanol extract, alkaloid and saponin-enriched fractions. Similar results were obtained when Jurkat T cells were exposed to the hexane leaf extract of *D. cauliflorum*, which was found to be non-toxic to the Jurkat T cells [38]. Mozonte et al. [39] also reported that the ethanol bark extract of *Q. insignis* and the methanol bark extract of *Canostelgia xalapensis* were non-toxic to both Balb/c mouse macrophages and human tumour cells. This is in agreement with the results obtained in this study, where *C. zeyheri* extracts were non-toxic to both Balb/c mouse peritoneal cells and Jurkat T cells, a human cancer cell line. Jurkat T cells, being transformed white blood cells may have been stimulated to grow in order to boost immunity just like the mouse peritoneal cells. The extracts from *C. zeyheri,* may, therefore, be contraindicated in leukaemic patients as they will probably encourage growth of the cancerous cells. The alkaloids, saponins and ethanol extract of *C. zeyheri* were not-toxic to Jurkat T cells and mouse peritoneal cells, but actually enhanced their growth. Extrapolation of the results to *in vivo* situation may suggest that whilst the plant extracts have antifungal effects, they may also increase the proliferation of the immune cells, thus providing enhanced fungicidal effects on the fungal pathogens. Although these extracts caused hemolysis of sheep erythrocytes, there was a large magnitude of difference between the IC_50 values_ and the hemolysis EC_50_ values_,_ implying a large therapeutic window.

## Conclusions

We conclude that the antifungal and non-toxic characteristics of the *C. zeyheri* ethanol extract, alkaloid, and saponin-enriched fractions makes these constituents strong candidates for the isolation of the antifungal active compounds. These compounds can then serve as templates in antifungal drug development after being modified to remove functional groups that could be responsible for causing haemolysis without altering the antifungal activity.

## Abbreviations

EC_50_, half maximal effective concentration (EC_50_) refers to the concentration of a drug, which induces a response halfway between the baseline and maximum after a specified exposure time; FBS, foetal bovine serum; HBSS, Hank’s Balanced Salt Solution; IC_50_, inhibition concentration that causes a 50 % reduction in enzyme activity; MTT, 3-(4,5-dimethylthiazol-2-yl)-2,5-diphenyltetrazolium bromide; SDA, Sabouraud dextrose agar; SDB, Sabouraud dextrose broth.
